# Auricular therapy for primary dysmenorrhea: A protocol for systematic review and meta-analysis of randomized controlled trials

**DOI:** 10.1097/MD.0000000000033382

**Published:** 2023-03-31

**Authors:** Qianhui Yu, Jiazhen Cao, Hongxiu Chen, Jing He, Xinyue Wang, Renming Liu, Tie Li

**Affiliations:** a Department of Acupuncture and Tuina, Changchun University of Chinese Medicine, Changchun, China.

**Keywords:** auricular therapy, meta-analysis, primary dysmenorrhea, protocol, systematic review

## Abstract

Primary dysmenorrhea (PD) is a common problem among women. It is defined as any degree of perceived cramping pain during menstruation without any evident pathology. Auricular therapy (AT), a widely used alternative medical treatment method as part of traditional Chinese acupuncture, lacks reliable evidence to support its safety and effectiveness for PD. We aimed to conduct a meta-analysis to investigate the efficacy and safety of AT in PD and to investigate possible factors impacting the particular efficacy of AT in PD by meta-regression.

**Methods:**

This protocol followed the PRISMA guidelines of the Preferred Reporting Items for Systematic Reviews and Meta-Analysis Protocols. The following 9 sources will be searched for randomized control trials of AT for PD: the Cochrane Central Register of Controlled Trials, PubMed, Medline, Embase, Web of Science, Chinese Biomedical Literature Database (CBM), China National Knowledge Infrastructure, Chinese Science and Technology Periodicals (VIP) database and WanFang Database from inception to January 1, 2023. Primary outcomes include visual rating scales and clinical efficacy rates, while secondary outcomes include endocrine hormone indicators related to PD and adverse events. Two reviewers will work independently on study selection, data extraction, and coding, including the risk of bias assessment in the included studies. While conducting a meta-analysis, Review Manager version 5.3 will be employed. Otherwise, a descriptive analysis will be performed. The results will be displayed as a risk ratio with 95% confidence intervals for dichotomous data as well as weight mean difference or standardized mean difference with 95% confidence intervals for continuous data.

**Results:**

This study’s protocol will investigate the efficacy and safety of AT in the treatment of PD in a systematic way.

**Conclusion:**

This systematic evaluation will objectively and systematically assess the efficacy and safety of AT in PD based on the available evidence, as well as provides clinicians with evidence to support the treatment of the disease.

## 1. Introduction

Dysmenorrhea is a painful uterine cramp caused by menstruation which can be divided into primary and secondary dysmenorrhea depending on the symptoms. Primary dysmenorrhea (PD) is pain that occurs in the absence of identifiable pelvic changes, and it is the most common gynecological condition affecting women.^[1,2]^ The clinical symptoms of pain are mainly due to excessive secretion of prostaglandins by the endometrium, which leads to excessive contraction of the uterus, which causes ischemia and hypoxia in the uterine muscles and consequently pain.^[3]^ The global prevalence of PD in childbearing women ranges from 45 to 95%, with 2 to 29% of them having severe pain. This has a significant impact on their overall quality of life, as well as their ability to work and attend school.^[4]^

Many women are used to seeing pain as a regular physical event, although it restricts daily life. For treatment, nonsteroidal anti-inflammatory drugs are used as first-line drugs for this condition which are also often used in combination with oral contraceptives.^[5,6]^ Although medications can relieve menstrual pain, they may also lead to adverse events such as digestive disorders, headaches, and drowsiness.^[7]^ Meanwhile, lifestyle changes and nonpharmacological treatments such as ear acupuncture, acupuncture, and regular exercise are considered to be complementary and alternative approaches to the treatment of PD.^[8]^

Auricular therapy (AT), one of the methods of Traditional Chinese Medicine, can regulate qi and blood by piercing or pressing specific points on the auricle to help achieve a balance of the yin and yang state of the organs and is suitable for treating a variety of diseases in the body.^[9]^ It was first mentioned in the earliest Chinese medical book, Huang di Neijing: The Yellow Emperor’s Classic of Internal Medicine, published >2000 years ago. In 1990, the World Health Organization recognized that AT regulates the functional systems of the whole body and positively affects the organism.^[10]^ A systematic review has shown that the application of auricular stimulation therapy is most effective in the treatment of dysmenorrhea. Still, its effectiveness and safety have not yet reached a definitive conclusion.^[11]^ With a high population of randomized control trials on AT for PD published and registered in recent years, it is essential to update the systemic review and synthesis more recent data to seek more consistent conclusions on the effectiveness of AT. As a result, a systematic review and meta-analysis of existing randomized control trials will be performed to show the clinical effectiveness and safety of ear point stimulation.

## 2. Methods

### 2.1. Protocol and registration

This agreement is registered on PROSPERO (registration number: CRD42022311680). The program will be carried out by the Cochrane Collaboration Handbook and the Preferred Reporting Items for Systematic Reviews and Meta-Analysis Protocols (PRISMA-P) declaration.^[12]^

### 2.2. Type of study

All randomized controlled trials on menstrual pain were included in the analysis, except for reports, observational cohort studies, case series, and reviews. In addition, Language and publication time are both unrestricted.

### 2.3. Types of participants.

We will include patients based on clinical diagnostic criteria, including pelvic examination and ultrasound scan showing no organic lesions. Even if they are suitable for PD, patients with identifiable pelvic pathology, pregnant women, and people with serious medical issues need to be excluded. There were no restrictions based on race, age, or nationality.

### 2.4. Interventions and comparators

Patients in the treatment group will be treated for PD primarily with interventions such as AT (including auricular acupuncture, ear point-based plasters, acupressure, electro-acupuncture of the ear, laser, and auricular bloodletting) which will also be considered in combination with other positive treatments. In the control group, we will include studies using Western medicine, no treatment, placebo, acupuncture, or other herbal medicines as a control intervention.

### 2.5. Types of outcome measures

(1) Primary outcomes include the following aspects:
  (a) The clinical effective rate;
  (b) Intensity of painful menstruation: the visual analog scale score.^[13]^
(2) Secondary outcomes include the following aspects:
  (a) Endocrine hormone indicators related to PD: such as prostaglandin F2α (PGF2α) and prostaglandin E2 (PGE2);
  (b) Adverse events (such as allergy, local infection, etc).

### 2.6. Search strategy

We will have 2 investigators working independently on PubMed, the Cochrane Central Register of Controlled Trials, Medline, Embase, and Web of Science, comprising 4 Chinese databases: Chinese Biomedical Literature Database (CBM), China National Knowledge Infrastructure, Chinese Science and Technology Periodicals (VIP) database, and WanFang Database. The search strategy included 3 components: clinical condition [“dysmenorrhea” or “primary dysmenorrhea” or “dysmenorrhea” or “ menstrual cramps” or “menstrual pain” or “ period pain” or “pelvic pain” or “painful menstruation” or “painful period” or “menstrual disorder” or “Uterine contraction” or “PD”], intervention (“auriculotherapy” or “auricular therapy “or “auricular stimulation” or “auricular plaster” or “ear acupressure” or “ electro-acupuncture of the ear “ or ‘auricular patch’ or ‘auricular acupuncture’ or” auricular bloodletting “ or “auricular*” or “ AT “ or “AA”) and study type (“randomized controlled trial” or “RCT” or “random allocation”). The above databases were searched. As shown in Table 1, the search strategy uses PubMed as an example.

**Table 1:** The search strategy for PubMed database.

Number	Search terms
#1	dysmenorrheas OR menstrual pain [MeSH]
#2	dysmenorrhea[Title/Abstract] OR dysmenorrhea [Title/Abstract] OR primary dysmenorrhea [Title/Abstract] OR menstrual cramps[Title/Abstract] OR menstrual pain[Title/Abstract] OR period pain[Title/Abstract] OR pelvic pain[Title/Abstract] OR painful menstruation [Title/Abstract] OR painful period[Title/Abstract] OR menstrual disorder[Title/Abstract] OR Uterine contraction[Title/Abstract] OR PD[Title/Abstract]
#3	#1 OR #2
#4	auriculotherapy [MeSH]
#5	auricular therapy[Title/Abstract] OR auricular stimulation[Title/Abstract] OR auricular plaster[Title/Abstract] OR ear acupressure[Title/Abstract] OR electro-acupuncture of the ear[Title/Abstract] OR auricular patch[Title/Abstract] OR auricular acupuncture[Title/Abstract] OR auricular bloodletting[Title/Abstract] OR auricular*[Title/Abstract] OR AT [Title/Abstract] OR AA[Title/Abstract]
#6	#4 OR #5
#7	randomized controlled trial [MeSH]
#8	randomized controlled trial [Publication Type] OR randomized controlled trial [All Fields] OR RCT [Title/Abstract] OR random allocation[Title/Abstract]
#9	#7 OR #8
#10	#3 AND #6 AND #9

Additional electronic databases will also use the same or adapted search strategy. In addition, the reference list of the included papers is searched manually, which expands the search of the electronic database. The last search update was conducted on January 1, 2023.

### 2.7. Data collection and analysis

#### 2.7.1. Selection of studies.

**Figure 1.**
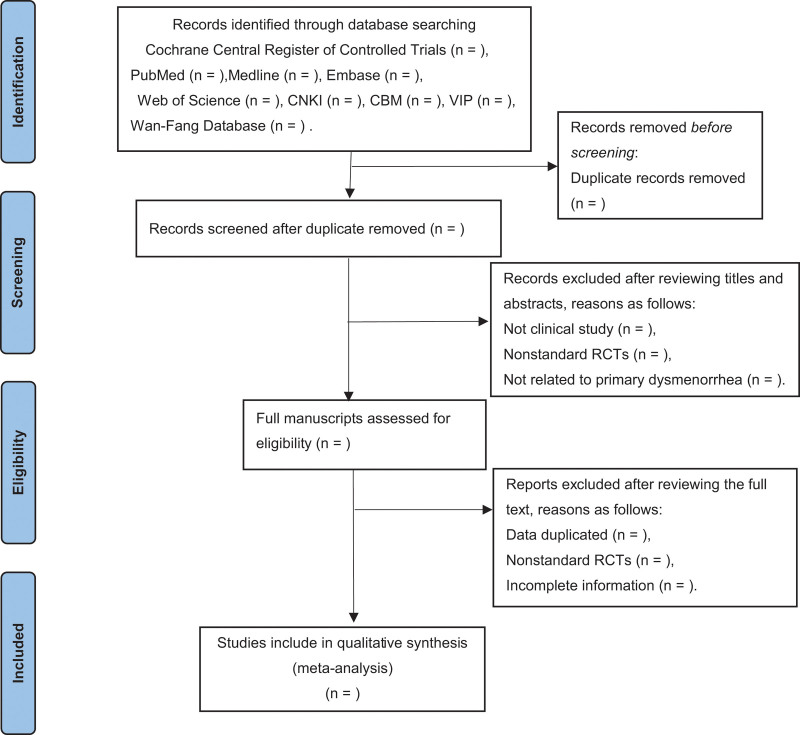
Flow diagram of study selection process.

There will be 2 researchers independently using Endnote software (Clarivate Analytics) for the selection of research literature. Firstly, we will make an initial selection by screening titles and abstracts. Secondly, we will download the full text of the relevant studies to make further selections based on inclusion criteria. All excluded studies will be listed in a table with the reasons for rejection. Disagreements about the data will be discussed through consensus when a third researcher joins. The detailed processing of the screening will be shown in the PRISMA flow chart (Fig. 1).

#### 2.7.2. Data extraction and management.

Information will be extracted from all eligible study data independently by 2 reviewers, including basic information (author, study title, journal and year of publication, etc), intervention information (treatment, duration, control group), outcomes, adverse effects, etc. Extracted data will be cross-checked by 2 extractors.

#### 2.7.3. Risk of bias assessment.

All included studies will be assessed for risk of bias according to the Cochrane Handbook V.5.1.0. risk of bias tool. We will primarily include the following items: random sequence generation, allocation concealment, blinding of participants and personnel, blinding of outcome assessments, incomplete outcome data, selective reporting, and other risks of bias. The above results will be assessed qualitatively using 3 levels low risk, high risk, and uncertain risk. During the assessment process, disagreements will be resolved through members’ discussion or consultation with other reviewers.

#### 2.7.4. Measures of treatment effect.

Review Manager 5.3 software (the Cochrane Collaboration, Oxford, England) will be used to calculate the outcome data. We will use 95% confidence intervals and risk ratios to estimate dichotomous data. For continuous outcomes, weighted mean differences or standardized mean differences will be used for comparison.

#### 2.7.5. Additions of missing data.

We will email the associated author for the essential information if an article has any missing or insufficient data. If no contact is established or the data is incomplete, the study will be excluded.

#### 2.7.6. Assessment of heterogeneity and data synthesis.

Heterogeneity was assessed using chi-squared tests and *I*^2^ values according to the statistical guidelines cited in the Cochrane Handbook.^[14]^ Data analyses were performed using either a fixed model with no significant heterogeneity or a random-effects model with considerable heterogeneity. *I*^2^ values of 50% and below suggested no statistically substantial between-study heterogeneity, so they were used in a fixed-effects model. Conversely (*I*^2^>50%), a random-effects model was used. If the data does not lend itself to quantitative analysis, we will conduct a descriptive analysis.

### 2.8. Subgroup analysis

Where there is significant heterogeneity in the study data, subgroup analyses will be conducted according to the severity and duration of dysmenorrhea, different types of ear therapy, different control groups, and outcomes measured.

### 2.9. Sensitivity analysis

We will use a study-by-study exclusion approach and then retake the remaining studies for sensitivity analysis to verify the robustness of the main results. For the analysis results, we will eliminate low-quality and highly heterogeneous studies to improve the accuracy and credibility of the results.

### 2.10. Reporting bias analysis

When the total number of included documents is 10 or over this number, we will pass on the symmetry of the funnel plot to determine publication and other reporting biases. In addition, Begg test and Egger test will be used to verify the symmetry of the funnel plot.

### 2.11. Confidence in cumulative evidence

Individual outcomes will be scored for general quality using the Grades of Recommendations for Assessment, Development, and Evaluation (GRADE pro) guideline development tool.^[15]^ The quality of evidence will be categorized into 4 levels: high, moderate, low, and very low quality based on the 5 GRADE criteria of study limitations, inconsistency, imprecision, indirectness, and risk of publication bias.

### 2.12. Ethics and dissemination

Ethics approval is not required because this is conducted on published data and not on individual patient information. The results will be submitted to a peer-reviewed journal and presented at relevant conferences.

### 2.13. Amendments

The information will be described in the final report, and if the protocol is modified.

## 3. Discussion

PD is mainly characterized by cyclical lower abdominal pain, as well as often accompanied by other symptoms such as dizziness, headaches, and nausea, which have a negative impact on life, work, and psychology.^[2,4,16]^ Although nonsteroidal anti-inflammatory drugs s can bring relief from painful symptoms. However, there are gastrointestinal and other side effects that make most patients reluctant to take the drug.^[17]^ Complementary and alternative therapies are gaining public acceptance due to their common side effects and high safety profile, and they will gradually become the mainstay of treatment for this disease. AT, as a traditional Chinese medicine external treatment, focuses on stimulating specific points on the ear through acupuncture, acupressure, and bloodletting to treat the disease.^[18–20]^ Its main components include auricular acupuncture, Vaccaria seeds, acupressure, electro-acupuncture of the ear, laser, and auricular bloodletting. Nowadays, it has been widely used by doctors in China to treat PD with AT.

Although a considerable number of clinical studies have been conducted to investigate the effectiveness of AT in PD, these studies have not been systematically summarized, and the overall evidence remains inconclusive. In this study, it is worthwhile to systematically evaluate the evidence for the effectiveness and safety of AT for PD and to provide recommendations for future research and practice in this area.

## Acknowledgment

We appreciate the financial support from the National Key R&D Program of China (No. 2021YFE0202900).

## Author contributions

**Conceptualization:** Qianhui Yu, Jiazhen Cao.

**Data curation:** Hongxiu Chen, Renming Liu.

**Formal analysis:** Hongxiu Chen, Jing He.

**Funding acquisition:** Tie Li.

**Investigation:** Qianhui Yu, Xinyue Wang.

**Methodology:** Qianhui Yu, Jiazhen Cao.

**Project administration:** Tie Li.

**Resources:** Qianhui Yu.

**Software:** Qianhui Yu, Jiazhen Cao.

**Supervision:** Tie Li.

**Validation:** Jing He.

**Visualization:** Renming Liu.

**Writing – original draft:** Qianhui Yu.

**Writing – review & editing:** Qianhui Yu, Tie Li.

## References

[1] Ferries-Rowe E Corey E Archer JS. Primary dysmenorrhea: diagnosis and therapy. Obstet Gynecol. 2020;136:1047–58. 33030880 10.1097/AOG.0000000000004096

[2] Kho KA Shields JK. Diagnosis and management of primary dysmenorrhea. JAMA. 2020;323:268–9. 31855238 10.1001/jama.2019.16921

[3] Guimarães I Póvoa AM. Primary dysmenorrhea: assessment and treatment. dismenorreia primária: avaliação e tratamento. Rev Bras Ginecol Obstet. 2020;42:501–7. 32559803 10.1055/s-0040-1712131 PMC10309238

[4] Karout S Soubra L Rahme D Karout L Khojah HMJ Itani R. Prevalence, risk factors, and management practices of primary dysmenorrhea among young females. BMC Womens Health. 2021;21:392. 34749716 10.1186/s12905-021-01532-w PMC8576974

[5] Kennedy S. Primary dysmenorrhoea. Lancet. 1997;349:1116. 9113008 10.1016/S0140-6736(05)63018-8

[6] Burnett M Lemyre M. Primary dysmenorrhea consensus guideline no. 345-Primary Dysmenorrhea Consensus Guideline. J Obstet Gynaecol Can. 2017;39:585–95. 28625286 10.1016/j.jogc.2016.12.023

[7] Oladosu FA Tu FF Hellman KM. Nonsteroidal antiinflammatory drug resistance in dysmenorrhea: epidemiology, causes, and treatment. Am J Obstet Gynecol. 2018;218:390–400. 28888592 10.1016/j.ajog.2017.08.108 PMC5839921

[8] López-Liria R Torres-Álamo L Vega-Ramírez FA. Efficacy of physiotherapy treatment in primary dysmenorrhea: a systematic review and meta-analysis. Int J Environ Res Public Health. 2021;18:7832. 34360122 10.3390/ijerph18157832 PMC8345570

[9] Suen LK Wong TK Leung AW. Is there a place for auricular therapy in the realm of nursing? Complement Ther Nurs Midwifery. 2001;7:132–9. 11855508 10.1054/ctnm.2001.0565

[10] Round R Litscher G Bahr F. Auricular acupuncture with laser. Evid Based Complement Alternat Med. 2013;2013:984763. 23935695 10.1155/2013/984763 PMC3710613

[11] Liu M Tong Y Chai L. Effects of auricular point acupressure on pain relief: a systematic review. Pain Manag Nurs. 2021;22:268–80. 32950391 10.1016/j.pmn.2020.07.007

[12] Liberati A Altman DG Tetzlaff J. The PRISMA statement for reporting systematic reviews and meta-analyses of studies that evaluate health care interventions: explanation and elaboration. PLoS Med. 2009;6:e1000100. 19621070 10.1371/journal.pmed.1000100 PMC2707010

[13] Reed MD Van Nostran W. Assessing pain intensity with the visual analog scale: a plea for uniformity. J Clin Pharmacol. 2014;54:241–4. 24374753 10.1002/jcph.250

[14] Higgins JPT Green S. Cochrane Handbook for Systematic Reviews of Interventions Version 5.1.0 (updated March 2011). The Cochrane Collaboration; 2011. Available at: www.cochrane-handbook.org.

[15] GRADEpro GDT. GRADEpro guideline development tool, 2015. Available at: https://gradepro.org.

[16] Jo J Lee SH. Heat therapy for primary dysmenorrhea: a systematic review and meta-analysis of its effects on pain relief and quality of life. Sci Rep. 2018;8:16252. 30389956 10.1038/s41598-018-34303-z PMC6214933

[17] Marjoribanks J Ayeleke RO Farquhar C Proctor M. Nonsteroidal anti-inflammatory drugs for dysmenorrhoea. Cochrane Database Syst Rev. 2015;2015:CD001751. 26224322 10.1002/14651858.CD001751.pub3 PMC6953236

[18] Wang Y Li SY Wang D. Transcutaneous auricular vagus nerve stimulation: from concept to application. Neurosci Bull. 2021;37:853–62. 33355897 10.1007/s12264-020-00619-y PMC8192665

[19] Hou PW Hsu HC Lin YW Tang NY Cheng CY Hsieh CL. The history, mechanism, and clinical application of auricular therapy in traditional Chinese medicine. Evid Based Complement Alternat Med. 2015;2015:495684. 26823672 10.1155/2015/495684 PMC4707384

[20] Gori L Firenzuoli F. Ear acupuncture in European traditional medicine. Evid Based Complement Alternat Med. 2007;4(Suppl 1):13–6. 18227925 10.1093/ecam/nem106 PMC2206232

